# Non-linear dose-response relationship between body mass index and stroke risk in middle-aged and elderly Chinese men: a nationwide Longitudinal Cohort Study from CHARLS

**DOI:** 10.3389/fendo.2023.1203896

**Published:** 2023-07-05

**Authors:** Gang Wei, Feng Lin, Changchun Cao, Haofei Hu, Yong Han

**Affiliations:** ^1^ Department of Emergency, Hechi People’s Hospital, Hechi, Guangxi Zhuang Autonomous Region, China; ^2^ Department of Rehabilitation, Shenzhen Dapeng New District Nan’ao People’s Hospital, Shenzhen, Guangdong, China; ^3^ Department of Nephrology, Shenzhen Second People’s Hospital, Shenzhen, Guangdong, China; ^4^ Department of Emergency, Shenzhen Second People’s Hospital, Shenzhen, Guangdong, China

**Keywords:** stroke, obesity, non-linear association, generalized additive model, smoothed curve fitting

## Abstract

**Objective:**

Body mass index (BMI) and stroke risk have been linked, but these findings are still debated. This study investigated the relationship between BMI and stroke risk in a middle-aged and elderly Chinese population.

**Methods:**

This study used four waves of CHARLS data (2011, 2013, 2015, and 2018), including 12,161 participants. The CHARLS sample was obtained by multi-stage probability sampling and all participants were assessed by one-to-one interviews using a standardized questionnaire. We used a Cox proportional-hazards regression model to examine the relationship between BMI and stroke risk. We used Cox proportional hazards regression with cubic spline functions and smooth curve fitting to identify the non-linear relationship between them. A series of sensitivity analyses were also conducted.

**Results:**

The multivariate Cox proportional hazards regression model identified a positive association between BMI and stroke risk (HR=1.025, 95% CI: 1.010-1.040). We also found a non-linear relationship between BMI and stroke incidence, with an inflection point at 26.63 kg/m^2^ for BMI. Each 1 kg/m^2^ increase in BMI to the left of the inflection point was related to a 4.4% increase in stroke risk (HR=1.044, 95% CI: 1.019-1.069). We stratified individuals by gender to further investigate their association and found a particular non-linear relationship and saturation effect between BMI and stroke risk in men, with the inflection point at 25.94 kg/m^2^. Each 1 kg/m^2^ increase in BMI to the left of the inflection point was related to a 7.6% increase in stroke risk (HR=1.076, 95% CI 1.034-1.119). The association was linear in women, with each 1 kg/m^2^ increase in BMI associated with a 2.1% increase in stroke risk (HR=1.021, 95% CI 1.002, 1.040).

**Conclusion:**

In men, there was a specific non-linear association and saturation effect of BMI with stroke (inflection point of 25.94 kg/m^2^), while in women, there was none. When males had a BMI below 25.94 kg/m^2^, the risk of stroke was significantly and positively associated with BMI. By controlling BMI below 25.94 kg/m^2^ in men, a further decrease in BMI may promote a significant reduction in stroke risk.

## Introduction

The definition of a stroke is an episode of acute neurological impairment thought to be brought on by hemorrhage or ischemia, lasting longer than 24 hours or until death (1). Stroke is a devastating disease with high morbidity, high mortality, adult disability, and poor treatment worldwide (2–4). According to the 2019 Global Burden of illness report, stroke-related illness burden increased from fifth in 1990 to third in 2019 (5). Furthermore, it is predicted that if nothing is done, there will be 7-8 million stroke fatalities worldwide by 2030 (6). The financial burden on families and society is enormous. Therefore, identifying risk factors for stroke and intervening in them is particularly important to prevent stroke and reduce the economic burden on society. Among the risk factors reported were high blood pressure, smoking, diabetes, obesity, and heart disease (7). As a major risk factor for heart disease, diabetes, hypertension, and other diseases, obesity is regarded as one of the most significant, controllable causes of stroke. Obesity is regarded as one of the most significant modifiable risk factors for stroke, as it also a high-risk factor for diabetes, hypertension, and heart disease (8, 9). Body mass index (BMI) is commonly considered an indicator of obesity, and clarifying the relationship between BMI and stroke risk is particularly important for stroke prevention and treatment and physician-patient communication.

However, the relationship between BMI and stroke risk is controversial and complicated. Several studies have found an association between BMI and incident stroke (10–13), while others have shown no relationship between them (14–16). In addition, another study has shown that low BMI is a risk factor for stroke (17). Therefore, we speculate that there may be a non-linear relationship between BMI and incident stroke. Unfortunately, most studies investigating the relationship between BMI and stroke have relied primarily on linear regression, and few cohort studies have investigated their non-linear association. In addition, these studies differed in terms of the implementation period, BMI range, sex ratio, and adjustment factors; thus, the relationship between BMI and the incidence of stroke in the Chinese population remains ambiguous. There are significant differences in body fat proportions and distribution patterns between men and women, and their relationship may vary across genders. We, therefore, propose the hypothesis that increased BMI may still be a risk factor for stroke, but the connection between them may be non-linear, and the association between them may vary across gender. We aimed to conduct a prospective cohort study using data from 2011 to 2018 based on the China Health and Retirement Longitudinal Study (CHARLS) to test this hypothesis and clarify the relationship between BMI and stroke risk in the Chinese population.

## Methods

### Study design

This cohort study was conducted using data from the CHARLS from 2011 to 2018 (18). BMI was the independent variable, and the outcome variable was incident stroke (dichotomous variables: stroke, non-stroke).

### Data source and study population

The CHARLS, an ongoing population-based, longitudinal cohort research that is now being conducted nationally to evaluate economic, social, and health status, provided the data for this study (18, 19). The CHARLS sample was obtained from 450 communities in 150 districts and 28 provinces through multi-stage probability sampling, with 10,257 households participating in the baseline survey and 17,708 participants. The sample was drawn from the Chinese population aged 45 or older at baseline (June 2011 to March 2012) (18, 19). All participants were assessed by one-on-one interviews using a standardized questionnaire. Participants underwent in-person follow-up every two years (18). The Biomedical Ethics Review Board of Peking University, China, approved the CHARLS study (IRB00001052-11015), and all participants provided written informed consent (18). This study’s data and related information can be downloaded from the CHARLS project website (http://charls.pku.edu.cn/).

**Figure 1:**
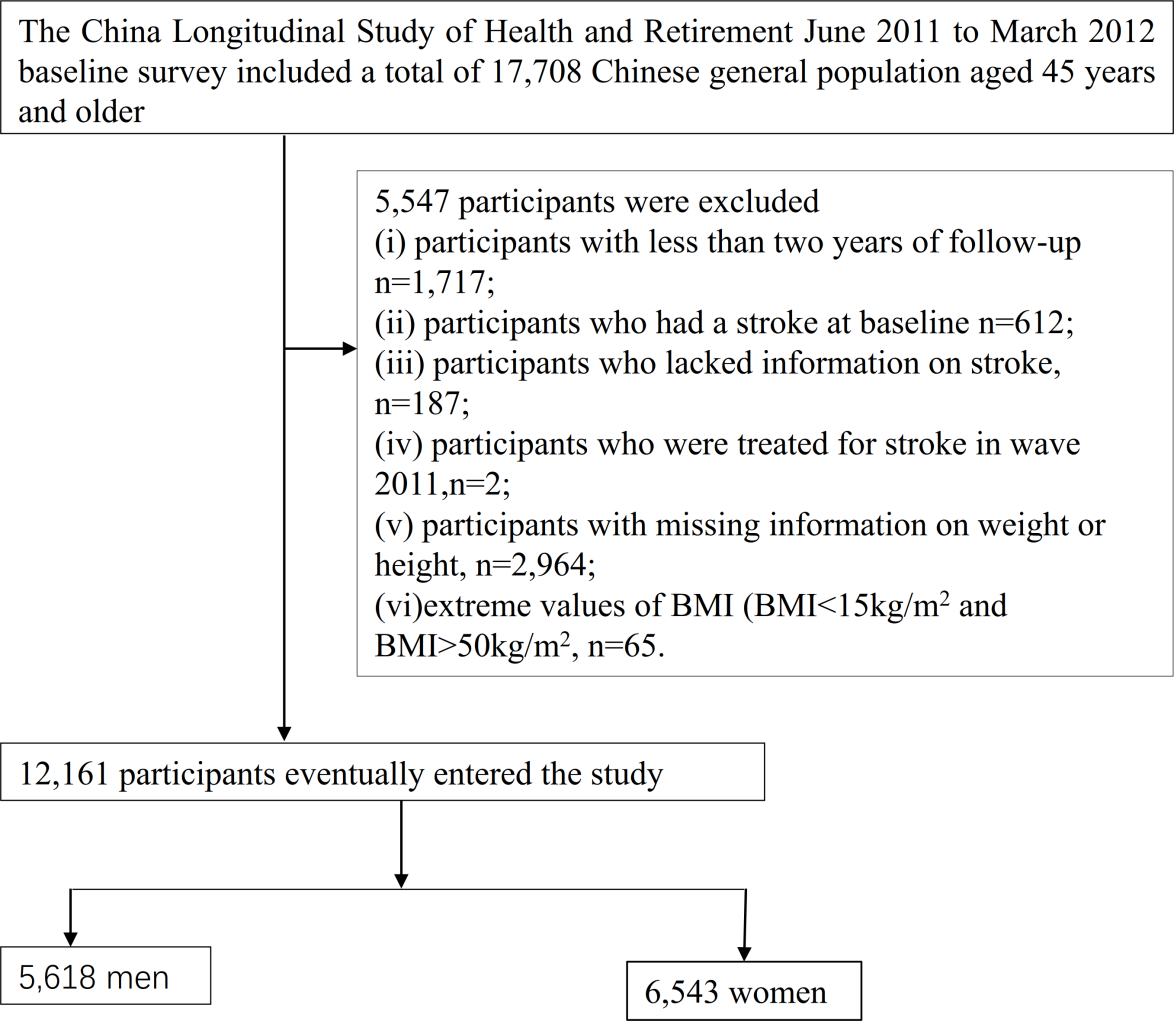
Flowchart of study participants.

Our study used data from four CHARLS waves (2011, 2013, 2015, and 2018). 17,708 participants were included in the baseline survey in 2011-2012. Firstly, we excluded participants with less than two years of follow-up (n=1,717). Secondly, we excluded participants who had a stroke at baseline (n=612) and who lacked information on stroke (n=187) or who were treated for stroke in wave 2011 (n=2). In addition, participants with missing weight data (n=2,900) and missing information on height (n= 64) and extreme values of BMI (BMI<15kg/m^2^ and BMI>50kg/m^2^, n=65) were also excluded (20). Finally, a total of 12,161 participants were included in the final analysis. The procedure for choosing participants is shown in **Figure 1**.

### Variables

#### Body mass index

The BMI is documented as a continuous variable. The procedure for precisely defining BMI is described below.BMI= weight/height^2^ (kg/m^2^). It is worth noting that information on weight and height was obtained at the 2011-2012 baseline survey. The categories of obesity (BMI>28 kg/m^2^), overweight (24<BMI ≤ 28kg/m^2^) and normal-weight (18.5<BMI ≤ 24kg/m^2^), underweight (BMI ≤ 18.5kg/m^2^) were established based on the definitions proposed by the China Obesity Working Group (21).

#### Diagnosis of stroke

Participants who did not have a history of stroke episodes at the baseline survey but reported a stroke experience at the follow-up were considered to have had a stroke event. As mentioned earlier (18, 19), information about stroke events was collected using standardized questions: (i) Were you told by your doctor that you had been diagnosed with a stroke? (ii) When were you first diagnosed/aware of the disease yourself? (iii) Are you currently receiving any follow-up treatment for your stroke? If the individual provided a positive response at follow-up, the respondent was classified as having a first diagnosis of stroke, and the self-reported time was recorded as the onset of the stroke. The time of the event was determined by subtracting the time of the baseline survey from the time of stroke onset. If the participant did not experience a stroke during any of the follow-up visits, the time of event onset was calculated as the time of the last survey minus the time of the baseline survey.

#### Covariates

Covariates were selected based on previous research and our clinical knowledge (18, 22–24). The subsequent factors were used as covariates: (i) categorical variables: diabetes mellitus (DM), sex, chronic lung disease(CLD), hypertension, smoking status, coronary heart disease (CHD), drinking status, chronic kidney disease(CKD), malignant tumors, mental disease; (ii) continuous variables: age, hematocrit (HCT), serum high-density lipoprotein cholesterol (HDL-c), hemoglobin concentration (HGB), total serum cholesterol (TC), blood urea nitrogen (BUN), platelet (PLT), diastolic blood pressure(DBP), serum triglyceride (TG), fasting plasma glucose (FPG), systolic blood pressure (SBP), Uric acid (UA), serum low-density lipoproteins cholesterol (LDL-c), hemoglobin A1c (HBA1c), serum creatinine (Scr).

#### Data collection

Interviewers were trained by CHARLS staff at Peking University to conduct interviews in respondents’ homes using the computer-assisted personal interview technique. The core CHARLS questionnaire included the following sections demographics, health status and functioning, physician-diagnosed chronic diseases, lifestyle, and health-related behaviors, including smoking status (never/ever/current), alcohol consumption status (never/ever/current), and physical activity (walking/moderate/vigorous). Structured questionnaire interviews were used to obtain self-reported physician diagnoses of CKD, hypertension, DM, CHD, CLD, and other diseases and the timing of diagnosis. Specific assessments were detailed in the CHARLS questionnaire and cohort profile (18). The interviewer also carried equipment to measure health functioning and performance in the respondent’s household, including height, weight, and blood pressure. Respondents were invited to the township hospital or local office of Disease Prevention and Control (CDC), where a trained nurse drew an 8 ml fasting blood sample. Testing for a complete blood count (CBC) was performed within 1-2 hours of sample collection. The remaining blood samples were separated into plasma and red blood cells and stored in a -20°C environment for transport while the CBC was performed. All blood samples were shipped back to Beijing for analysis at the CDC (18).

#### Missing data handling

In our study, there were 5 (0.04%), 39 (0.32%), 40(0.35%), 42 (5.23%), 46 (0.38%), 51 (0.42%), 60 (4.9%),105 (0.86%), 3085(25.37%), 3168 (26.05%), 3224 (26.51%), 3224 (26.51%), 3224(26.51%), 3225 (26.52%), 3230 (26.56%), 3231 (26.57%), 3241(26.65%), 3243 (26.67%), 3245 (26.68%), 3266 (26.86%), and 3268 (26.87%) participants with missing data for drinking status, smoking status, CLD, mental disease, malignant tumors, CHD, CKD, DM, HCT, HBA1c, BUN, CRP, UA, HDL-c, TG, TC, FPG, LDL-c, SCR, PLT, and HGB respectively. In order to reduce the deviation caused by missing variables, which makes it impossible to accurately depict the statistical efficacy of the target sample during the modeling phase, multiple imputations are used for the missing data in this study (25, 26). Age, gender, drinking status, smoking status, CLD, mental disease, CHD, CKD, DM, HCT, HBA1c, BUN, CRP, UA, HDL-c, TG, TC, FPG, LDL-c, SCR, malignant tumors, PLT, and HGB were all taken into account in the imputation model (Iterations were 10, and the kind of regression was linear). Processes for missing data analysis employ the assumptions of missing-at-random (MAR) (25).

### Statistical analysis

The means and standard deviations were reported for continuous variables with Gaussian distributions. Medians were reported for skewed distributions, while frequencies and percentages were reported for categorical variables. To examine if certain BMI groups differed from one another, we employed the Kruskal-Wallis H test (skewed distribution), the One-Way ANOVA test (normal distribution), or the χ2 test (categorical variables).

Three distinct models were constructed using univariate and multivariate Cox proportional hazards regression models to examine the association between BMI and the risk of stroke in males, females, and all participants. These models included: (i) crude model: no covariates were adjusted; (ii) Model I: adjusted for age, sex, and drinking, smoking; and Model II: adjusted for age, sex, CRP, HGB, LDL-c, HDL-c, TG, HBA1c, FPG, HCT, HBA1c, hypertension, diabetes, SBP, diabetes, CKD, CHD, mental disease, CLD. The calculated effect sizes were reported with 95% confidence intervals (95%CI). Adjustments for confounding variables were made based on clinical knowledge and published reports (12, 27–29). Note that in the male and female subgroups, neither Model I nor Model II included sex among the variables that were adjusted for. In addition, the final multivariate Cox proportional hazards regression equation did not include TC due to its collinearity with other variables (**Supplementary Table S1**).

We also examined the non-linear relationship between BMI and stroke risk in men, women, and all participants using Cox proportional hazards regression with cubic spline function and smoothed curve fitting. We first utilized a recursive technique to locate the inflection point if a non-linear relationship was discovered. A two-piecewise Cox proportional hazards regression model was then constructed on both sides of the inflection point. The best model to explain the connection between BMI and the risk of stroke was ultimately selected using a log-likelihood ratio test. In addition, we performed subgroup analyses. We converted age into categorical variables (age: <60, 60 to <70, ≥70 years) and used Cox proportional hazards regression with cubic spline functions and smooth curve fitting to identify the non-linear relationship between BMI and stroke risk in different age subgroups.

To assess the validity of our findings, we conducted multiple sensitivity analyses. According to the definition provided by the Chinese Obesity Task Force, we transformed BMI into a categorical variable. We calculated P for the trend to validate the finding of BMI as a continuous variable and to explore the possibility of a non-linear association between BMI and stroke risk. Since smoking, hypertension, and diabetes are strongly associated with stroke (30–33), We explored the association between BMI and stroke in men, women, and the entire sample by excluding smokers, hypertensive patients, or diabetic patients, respectively, from sensitivity analyses. Furthermore, we investigated potential unobserved confounders between BMI and stroke risk by calculating E-values (34).

All of the results were made in line with the STROBE statement (35). R statistical software tools (http://www.r-project.org, The R Foundation) and Empower Stats (X&Y Solutions, Inc., Boston, MA, http://www.empowerstats.com) were used for all analyses. The statistical significance was set at P values lower than 0.05(two-sided).

## Results

### Participants’ characteristics

**Figure 2:**
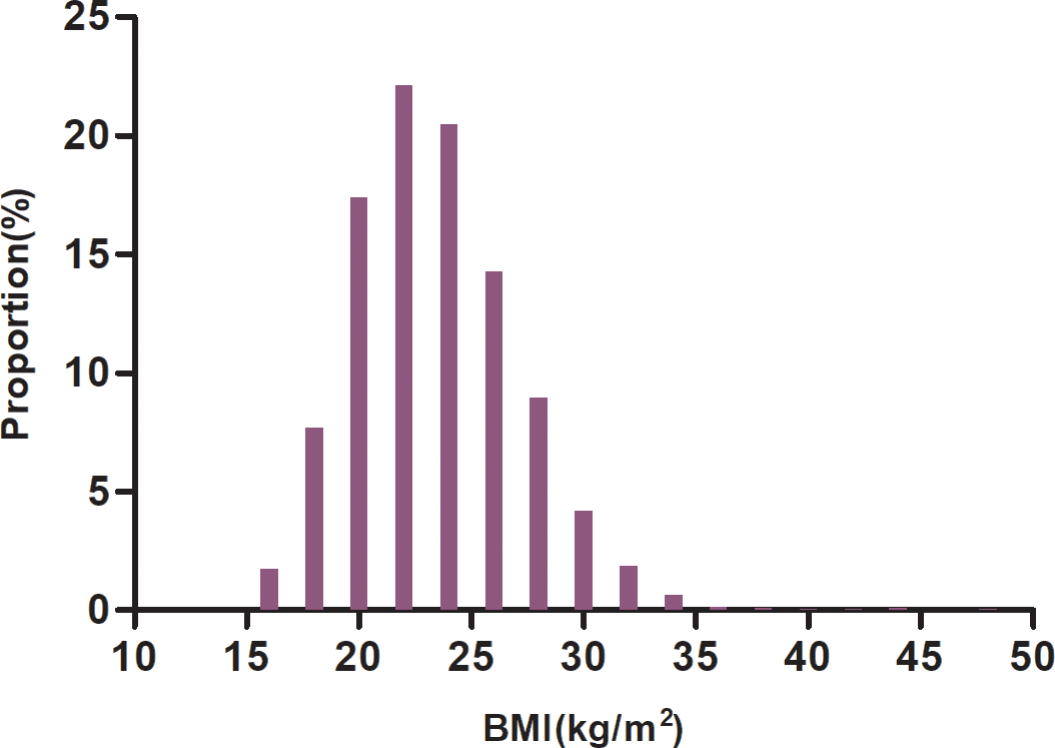
Distribution of BMI. **Figure 2**. BMI presented a normal distribution, with a mean of 23.45kg/m^2^.

The demographic and clinical characteristics of the participants in the study are detailed in **Table 1**. There were 12,161 participants in the final analysis, and 46.20% were males. Of these, 1,335 participants had a stroke, with a cumulative incidence of 10.98%. BMI presented a normal distribution, with a mean of 23.45kg/m^2^ (**Figure 2**). We divided participants into four subgroups according to the definition proposed by the Chinese Obesity Task Force. In comparison to the underweight group, PLT, TC, FPG, TG, CPR, LDL-c, HBA1C, UA, HCT, HGB, SBP, and DBP increased significantly in the obese group, while the inverse was true for age, HDL-c, BUN, and Scr. In addition, the proportion of never-smokers, women, CHD, malignant tumors, and diabetes was greater in the obese group.

**Table 1:** The baseline characteristics of participants.

BMI group (kg/m^2^)	Underweight (<18.5)	Normal-weight (18.5-24)	Over-weight (24-28)	Obesity (≥28)	P-value
Participants (n)	800	6490	3524	1347	
Age (years, mean ± SD)	64.69 ± 10.48	59.32 ± 9.70	57.45 ± 9.06	56.67 ± 8.77	<0.001
PLT (10^9/L, mean ± SD)	210.27 ± 70.93	210.37 ± 73.68	212.10 ± 71.83	218.98 ± 71.43	0.001
BUN (mmol/L, mean ± SD)	16.44 ± 5.67	15.81 ± 4.58	15.51 ± 4.35	15.27 ± 4.38	<0.001
FPG (mg/L, mean ± SD)	105.48 ± 34.95	107.48 ± 35.59	112.98 ± 37.59	116.73 ± 40.38	<0.001
Scr (mg/dL, mean ± SD)	0.79 ± 0.28	0.78 ± 0.25	0.77 ± 0.20	0.76 ± 0.20	0.003
TC (mg/dL, mean ± SD)	187.96 ± 35.28	190.99 ± 36.12	196.74 ± 38.18	200.22 ± 40.16	<0.001
TG (mg/dL, median, quartile)	88.50 (62.83-126.56)	99.12 (67.26-150.70)	125.67 (84.07-189.32)	148.68 (100.89-216.38)	<0.001
HDL-c (mg/dL, mean ± SD)	58.58 ± 15.53	53.66 ± 15.34	47.93 ± 13.73	44.18 ± 12.86	<0.001
LDL-c (mg/dL, mean ± SD)	110.31 ± 32.24	114.15 ± 33.73	119.09 ± 35.74	119.16 ± 37.76	<0.001
CRP (mg/L, median, quartile)	0.91 (0.39-3.42)	0.92 (0.42-2.71)	1.22 (0.55-2.89)	1.70 (0.76-3.95)	0.013
HBA1C (%,mean ± SD)	5.15 ± 0.73	5.19 ± 0.75	5.32 ± 0.85	5.44 ± 0.88	<0.001
UA (mg/dL, mean ± SD)	4.27 ± 1.26	4.37 ± 1.24	4.51 ± 1.23	4.62 ± 1.24	<0.001
HCT (%,mean ± SD)	40.13 ± 6.37	41.23 ± 6.30	41.77 ± 6.33	41.95 ± 6.06	<0.001
HGB (g/L, mean ± SD)	13.66 ± 2.10	14.27 ± 2.20	14.40 ± 2.26	14.54 ± 2.27	<0.001
SBP (mmHg, mean ± SD)	126.46 ± 30.38	128.63 ± 28.52	133.37 ± 28.16	139.47 ± 36.94	<0.001
DBP (mmHg, mean ± SD)	70.25 ± 13.73	73.32 ± 13.11	77.47 ± 13.14	79.78 ± 15.22	<0.001
Sex					<0.001
Male	375 (46.88%)	3380 (52.08%)	1423 (40.38%)	440 (32.67%)	
Female	425 (53.12%)	3110 (47.92%)	2101 (59.62%)	907 (67.33%)	
Diabetes (n.%)	18 (2.25%)	225 (3.47%)	260 (7.38%)	148 (10.99%)	<0.001
Malignant tumors (n.%)	11 (1.38%)	49 (0.76%)	45 (1.28%)	20 (1.48%)	0.014
Hypertension (n.%)	91 (11.38%)	1050 (16.18%)	1004 (28.49%)	597 (44.32%)	<0.001
CLD (n, %)	150 (18.75%)	665 (10.25%)	308 (8.74%)	118 (8.76%)	
CHD (n, %)	72 (9.00%)	581 (8.95%)	451 (12.80%)	236 (17.52%)	<0.001
CKD (n, %)	63 (7.88%)	418 (6.44%)	211 (5.99%)	71 (5.27%)	0.086
Mental disease (n, %)	8 (1.00%)	71 (1.09%)	47 (1.33%)	12 (0.89%)	0.535
Smoking status (n, %)					<0.001
Never	444 (55.50%)	3630 (55.93%)	2380 (67.54%)	988 (73.35%)	
Ever	65 (8.12%)	509 (7.84%)	327 (9.28%)	121 (8.98%)	
Current	291 (36.38%)	2351 (36.22%)	817 (23.18%)	238 (17.67%)	
Drinking status					<0.001
Never	103 (12.88%)	926 (14.27%)	444 (12.60%)	171 (12.69%)	
Ever	514 (64.25%)	3759 (57.92%)	2283 (64.78%)	946 (70.23%)	
Current	183 (22.88%)	1805 (27.81%)	797 (22.62%)	230 (17.07%)	

SD, standard deviation; n, number (%); BMI, body mass index; PLT, platelet; TG, triglyceride; HGB, hemoglobin concentration; TC, total cholesterol; HCT, hematocrit; LDL-c, low-density lipoproteins cholesterol; Scr, serum creatinine; HDL-c, high-density lipoprotein cholesterol; CKD, Chronic kidney diseases; BUN, blood urea nitrogen; ALT, alanine aminotransferase; AST, aspartate aminotransferase; CLD, Chronic Lung Diseases; CHD, coronary heart disease.

### Relationship between BMI and the risk of stroke in all participants

To investigate the connection between BMI and stroke risk across all individuals, three models were constructed using Cox proportional hazards regression models(**Table 2**). 1 kg/m^2^ rise in BMI was linked to a 5.8% increase in the risk of stroke in the crude model (HR=1.058, 95% CI 1.045-1.072). The incidence of stroke increased by 6.6% for every kg/m^2^ rise in BMI in Model I (HR=1.066, 95% CI 1.053-1.079, P0.001). Each extra kg/m^2^ of BMI in Model II was associated with a 2.5% increase in stroke risk(HR=1.025, 95%CI 1.010-1.040).

**Table 2:** Relationship between BMI and the risk of stroke in men, women, and all participants.

	Exposure	Crude model (HR, 95%CI) P	Model I (HR, 95%CI) P	Model II (HR, 95%CI) P
**All**	BMI (kg/m^2^)	1.058 (1.045, 1.072) <0.001	1.066 (1.053, 1.079) <0.001	1.025 (1.010, 1.040) <0.001
BMI group				
	Underweight	Ref	Ref	Ref
	Normal	1.198 (0.915, 1.568) 0.189	1.438 (1.096, 1.886) 0.009	1.261 (0.959, 1.658) 0.096
	Overweight	1.636 (1.245, 2.151) <0.001	2.061 (1.563, 2.719) <0.001	1.434 (1.079, 1.907) 0.013
	Obesity	2.175 (1.630, 2.903) <0.001	2.771 (2.068, 3.713) <0.001	1.546 (1.137, 2.103) 0.006
	P for trend	<0.001	<0.001	<0.001
**Male**	BMI (kg/m^2^)	1.066 (1.044, 1.088) <0.00001	1.078 (1.056, 1.100) <0.001	1.032 (1.007, 1.057) 0.011
	BMI group			
	Underweight	Ref	Ref	Ref
	Normal	1.231 (0.812, 1.866) 0.32758	1.449 (0.954, 2.201) 0.08215	1.277 (0.838, 1.947) 0.25515
	Overweight	1.839 (1.202, 2.815) 0.00501	2.308 (1.500, 3.553) 0.00014	1.576 (1.011, 2.456) 0.04483
	Obesity	2.133 (1.336, 3.407) 0.00151	2.750 (1.708, 4.427) 0.00003	1.425 (0.865, 2.350) 0.16468
	P for trend	<0.001	<0.001	<0.001
**Female**	BMI (kg/m^2^)	1.051 (1.034, 1.069) <0.00001	1.059 (1.042, 1.076) <0.00001	1.021 (1.002, 1.040) 0.03028
BMI group				
	Underweight	Ref	Ref	Ref
	Normal	1.194 (0.838, 1.701) 0.32632	1.463 (1.024, 2.090) 0.03674	1.269 (0.884, 1.821) 0.19726
	Overweight	1.502 (1.051, 2.147) 0.02541	1.934 (1.347, 2.777) 0.00035	1.365 (0.940, 1.983) 0.10252
	Obesity	2.133 (1.473, 3.089) 0.00006	2.760 (1.898, 4.015) <0.00001	1.614 (1.086, 2.399) 0.01788
	P for trend	<0.001	<0.001	<0.001

Crude mode1, we did not adjust other covariates.

Model I, we adjusted age, sex, drinking status, and smoking status.

Model II, we adjusted age, sex, CRP, HGB, LDL-c, HDL-c, TG, FPG, HCT, HBA1c, hypertension, diabetes, SBP, CKD, CHD, mental disease, CLD, drinking status, smoking status.

In the male and female subgroups, Models I and II were not adjusted for the stratification variable sex.

Besides, we reintroduced the categorically transformed BMI into the model after converting it from a continuous variable to a categorical variable. Using underweight participants as a reference, the multivariate-adjusted model’s findings revealed an HR of 1.261 (95% CI: 0.959-1.658) for participants with normal weight, 1.434 (95% CI: 1.079-1.907) for overweight participants, and 1.546(95% CI: 1.137- 2.103) for obese participants (**Table 2**).

### Association between BMI and stroke risk based on gender stratification.

We also used Cox proportional hazards regression models to construct three models to examine the relationship between BMI and stroke risk in men and women. According to the multivariate-adjusted model’s findings, a 1 kg/m^2^ rise in BMI was linked to a 5.8% higher risk of stroke in male participants (HR=1.032, 95% CI 1.007-1.057). Using underweight participants as a reference, the HR was 1.277 (95% CI: 0.838-1.947) for normal BMI participants, 1.576 (95% CI: 1.011-2.456) for overweight participants, and 1.425 (95% CI 0.865- 2.350) for obese participants (**Table 2**).

In female participants, results from multivariate-adjusted models showed that a 1 kg/m^2^ increase in BMI was associated with a 2.1% increase in stroke risk (HR=1.021, 95% CI 1.002-1.040, p=0.03). Using underweight participants as a reference, the HR was 1.269 (95% CI: 0.884-1.821) for normal BMI participants, 1.365 (95% CI: 0.940-1.983) for overweight participants, and 1.614 (95% CI 1.086-2.399) for obese participants (**Table 2**).

### Non-linear relationships addressed by Cox proportional hazards regression with cubic spline functions and smooth curve fitting

Multivariate adjusted models based on all participants showed no significant difference in the risk of stroke in normal-weight participants compared to underweight participants (p>0.05). In contrast, overweight and obese participants had a significantly increased risk of stroke and had similar HR values. These results suggest that there may be a non-linear association between BMI and stroke.

**Figure 3:**
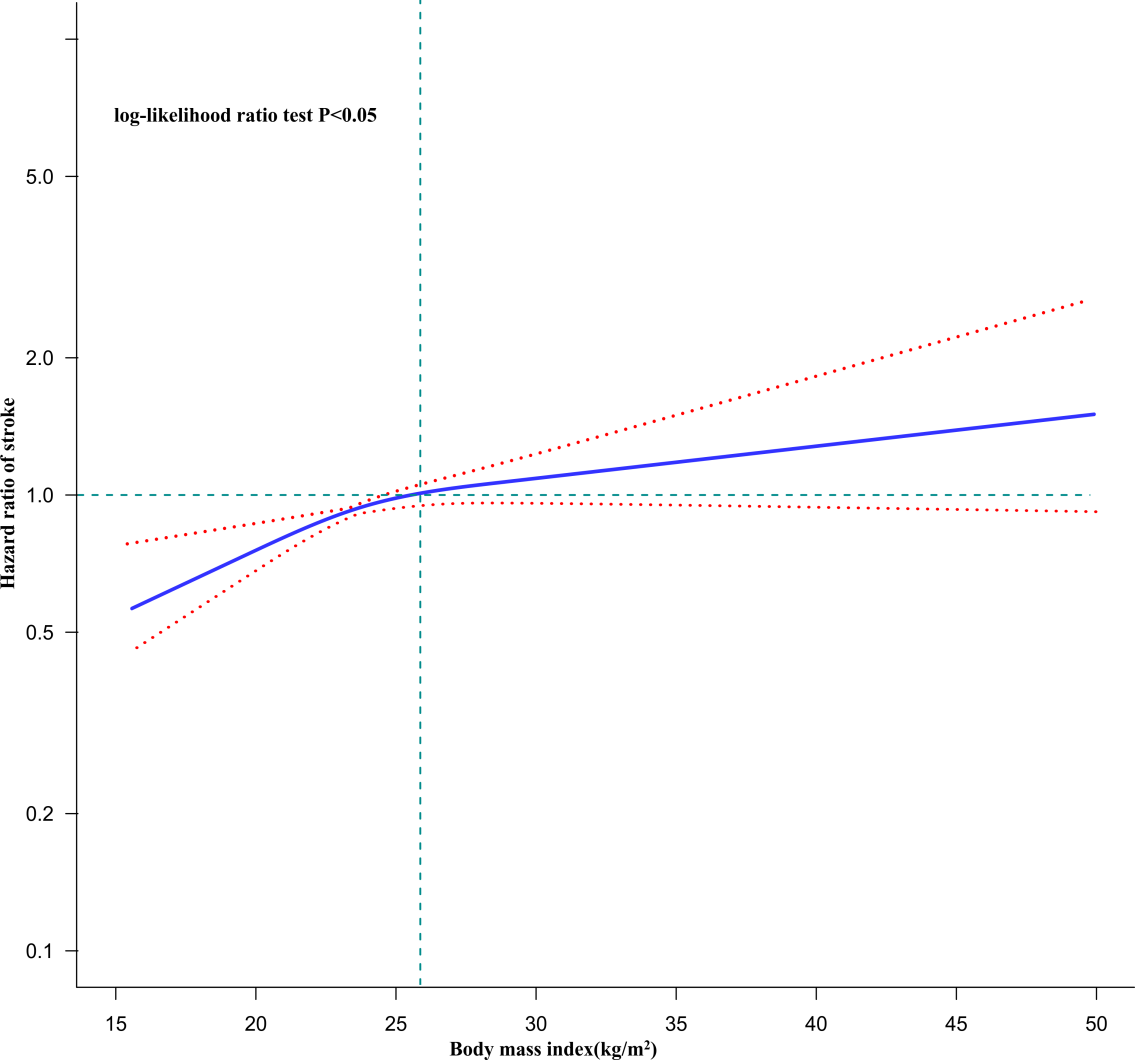
The non-linear relationship between BMI and the risk of stroke in all participants. **Figure 3**. A non-linear relationship was detected after adjusting for age, sex, CRP, HGB, LDL-c, HDL-c, TG, FPG, HCT, HBA1c, hypertension, diabetes, SBP, CKD, CHD, mental disease, CLD, drinking status, smoking status.

Using Cox proportional hazards regression with cubic spline functions and smooth curve fitting, we discovered that the association between BMI and stroke incidence was non-linear (**Figure 3**), with a log-likelihood ratio test of P less than 0.05. We adjusted for variables including age, sex, CRP, HGB, LDL-c, HDL-c, TG, FPG, HCT, HBA1c, hypertension, diabetes SBP, CKD, CHD, mental illness, CLD, drinking status, and smoking status. Using a recursive technique, we first identified a BMI inflection point of 26.63 kg/m^2^. Then we used a two-slice Cox proportional-hazards regression model to determine effect sizes and confidence intervals for the left and right sides of the inflection point. Each 1 kg/m^2^ increase in BMI was linked to a 4.4% increase in the incidence of stroke on the left side of the inflection point (HR=1.044, 95% CI 1.019-1.069, p=0.002). The HR and 95% CI on the right side of the inflection point were 0.998 and 0.967,1.030, respectively, but there was no significant difference (**Table 3**).

**Table 3:** The results of the two-piecewise linear regression model.

	Male (HR, 95%CI, P)	Female (HR, 95%CI, P)	All participants (HR, 95%CI, P)
Fitting model by standard linear regression	1.032 (1.007, 1.057) 0.011	1.021 (1.002, 1.040) 0.030	1.025 (1.010, 1.040) 0.001
Fitting model by two-piecewise linear regression			
Inflection points of BMI (kg/m^2^)	25.94	30.59	26.63
≤ Inflection point	1.076 (1.034, 1.119) <0.001	1.030 (1.007, 1.055) 0.012	1.044 (1.019, 1.069) <0.001
> Inflection point	0.973 (0.920, 1.029) 0.334	0.976 (0.906, 1.051) 0.517	0.998 (0.967, 1.030) 0.895
P for log-likelihood ratio test	0.008	0.179	0.047

Note1, In all participants, we adjusted age, sex, CRP, HGB, LDL-c, HDL-c, TG, FPG, HCT, HBA1c, hypertension, diabetes, SBP, CKD, CHD, mental disease, CLD, drinking status, smoking status.

Note 2, There was no adjustment for sex in the male and female subgroups.

### Non-linear association between BMI and the risk of stroke in gender subgroups

**Figure 4:**
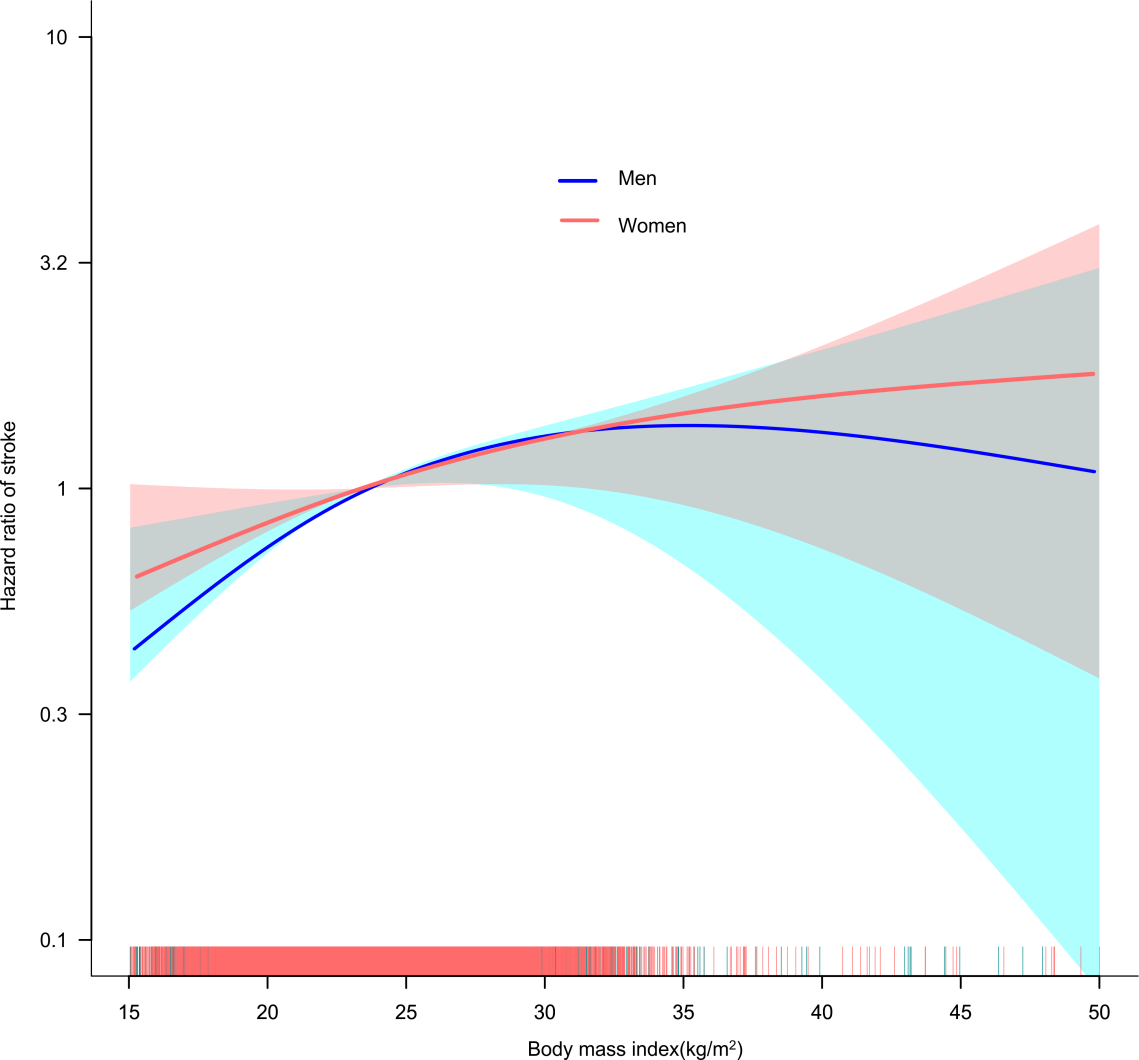
The non-linear relationship between BMI and the risk of stroke in men and women. A non-linear relationship was detected after adjusting for age, CRP, HGB, LDL-c, HDL-c, TG, FPG, HCT, HBA1c, hypertension, diabetes, SBP, CKD, CHD, mental disease, CLD, drinking status, smoking status. In contrast, in women the non-linear relationship between BMI and stroke risk did not establish.

We discovered that the association between BMI and stroke risk in men was non-linear (**Figure 4**, log-likelihood ratio test P<0.05). The inflection point for BMI was 25.94 kg/m2, and each 1 kg/m2 increase in BMI was associated with a 7.6% increase in the risk of stroke on the right before the inflection point (HR=1.076, 95% CI: 1.034-1.119, P<0.001). Their relationship on the left side of the inflection point was not statistically significant in men. In contrast, the non-linear association between BMI and stroke risk did not exist in females (log-likelihood ratio test P > 0.05) (**Table 3**).

### Non-linear association between BMI and the risk of stroke in age subgroups

Using the same method, we found that the association between BMI and stroke risk was non-linear in all age subgroups (log-likelihood ratio test P<0.05). Adjusted variables included age, sex, CRP, HGB, LDL-c, HDL-c, TG, FPG, HCT, HBA1c, hypertension, diabetes, SBP, CKD, CHD, mental illness, CLD, drinking status, and smoking status. On the less-than-inflection point side, the HRs for BMI and stroke risk were 1.065 (1.027, 1.104), 1.057 (1.023, 1.092), and 0.643 (0.449, 0.920) for the age <60, 60 to <70, and ≥70 subgroups, respectively. Their relationship on the side greater than the inflection point was not statistically significant in all age subgroups. (**Supplementary Table S3**).

### Sensitivity analysis

We conducted sensitivity analyses on participants without diabetes (n=11,510). After adjusting for confounding variables (including age, sex, CRP, HGB, LDL-c, HDL-c, TG, FPG, HCT, HBA1c, hypertension, SBP, CKD, CHD, mental disease, CLD), two-stage linear regression model analysis revealed a non-linear relationship between BMI and stroke risk, while in women participants, there was no non-linear relationship between them. Besides, we also excluded participants with hypertension (n=9,419). After adjusting for confounding variables (including age, sex, CRP, HGB, LDL-c, HDL-c, TG, HBA1c, FPG, HCT, diabetes, SBP, CKD, CHD, mental disease, CLD), similar results were obtained, that is, the relationship between BMI and stroke risk was non-linear in male participants and their relationship was not statistically significant in female participants. In addition, we also excluded participants with CKD (n=11,398) for sensitivity analysis and found that their relationship remained non-linear in male participants and linear in females (adjusted age, sex, CRP, HGB, LDL-c, HDL-c, TG, HBA1c, FPG, HCT, HBA1c, hypertension, SBP, CKD, CHD, mental disease, CLD) (**Table 4**). To evaluate the sensitivity to unmeasured confounders, an E-value was also calculated. The E-value (1.31) was higher than the relative risk of BMI and unmeasured confounders (1.27), suggesting that unknown or unmeasured factors may not have significantly affected the link between BMI and stroke risk. It is obvious that our results are reliable based on all sensitivity assessments.

**Table 4:** Relationship between BMI and the risk of stroke analyzed by two-piecewise linear regression model in different sensitivity analyses.

The risk of stroke	Male (HR, 95%CI, P)	Female (HR, 95%CI, P)	All participants (HR, 95%CI, P)
Model I:			
Standard linear regression model	1.034 (1.008, 1.061) 0.009	1.021 (1.001, 1.042) 0.040	1.026 (1.010, 1.042) 0.001
Fitting model by two-piecewise linear regression			
Inflection point of BMI (kg/m^2^)	25.86	30.41	27.27
≤ Inflection point	1.078 (1.034, 1.123) <0.001	1.032 (1.006, 1.058) 0.014	1.044 (1.020, 1.069) <0.001
> Inflection point	0.977 (0.922, 1.035) 0.428	0.971 (0.897, 1.052) 0.476	0.993 (0.957, 1.031) 0.729
P for log-likelihood ratio test	0.012	0.164	0.044
Model II:			
Standard linear regression model	1.038 (1.005, 1.073) 0.0257	1.027 (1.001, 1.053) 0.0438	1.030 (1.010, 1.051) 0.004
Fitting model by two-piecewise linear regression			
Inflection point of BMI (kg/m^2^)	22.482	29.584	28.981
≤ Inflection point	1.145 (1.052, 1.248) 0.002	1.042 (1.009, 1.076) 0.013	1.046 (1.020, 1.073) <0.001
> Inflection point	0.989 (0.937, 1.044) 0.692	0.960 (0.866, 1.065) 0.443	0.963 (0.890, 1.042) 0.347
P for log-likelihood ratio test	0.011	0.130	0.043
Model III:			
Fitting model by standard linear regression	1.028 (1.002, 1.054) 0.037	1.021 (1.001, 1.042) 0.036	1.023 (1.007, 1.039) 0.004
Fitting model by two-piecewise linear regression			
Inflection point of BMI (kg/m^2^)	26.223	19.996	27.11
≤ Inflection point	1.069 (1.027, 1.114) 0.001	1.082 (0.943, 1.241) 0.263	1.040 (1.015, 1.065) <0.001
> Inflection point	0.966 (0.908, 1.027) 0.268	1.017 (0.995, 1.040) 0.126	0.996 (0.962, 1.032) 0.826
P for log-likelihood ratio test	0.011	0.398	0.075

Model I was a sensitivity analysis in participants without diabetes. We adjusted age, sex, CRP, HGB, LDL-c, HDL-c, TG, FPG, HCT, HBA1c, hypertension, SBP, CKD, CHD, mental diaease, CLD, drinking status, smoking status.

Model II was a sensitivity analysis conducted on non-hypertension participants. We adjusted age, sex, CRP, HGB, LDL-c, HDL-c, TG, FPG, HCT, HBA1c, diabetes, SBP, CKD, CHD, mental diaease, CLD, drinking status, smoking status.

Model III was a sensitivity analysis conducted on participants without CKD. We adjusted age, sex, CRP, HGB, LDL-c, HDL-c, TG, FPG, HCT, HBA1c, hypertension, diabetes, SBP, CHD, mental diaease, CLD, drinking status, smoking status.

Note 2, There was no adjustment for sex in the male and female subgroups.

OR, odds ratios; CI, confidence; Ref: reference

## Discussion

The present study sought to investigate the association between BMI and stroke risk. In male participants, we discovered a non-linear rather than a linear link between them. The connection between BMI and stroke risk in males was a saturated effect curve with a point of inflection at 25.94 kg/m^2^. In women, however, the relationship between BMI and incident stroke was linear.

The results of many studies in the past have shown that obesity is a high-risk factor for stroke (22, 36, 37). However, the association between BMI and the risk of stroke is still uncertain and controversial. In a Chinese cohort study of 3,500 hypertensive patients aged ≥60 years, the result of multivariate Cox regression showed a positive association between BMI and the incidence of new stroke after adjusting for potential confounders (HR=1.14; 95% CI: 1.05-1.34; P=0.005) (13). Another cohort study of 154,736 adults showed that the relative risk (95% CI) of stroke compared to normal weight (BMI 18.5-24.9) participants were 0.86 (0.80-0.93) for those who were underweight (BMI 25-29.9kg/m^2^), 1.43 (1.36-1.52) for those who were overweight (BMI 25-29.9kg/m^2^), and 1.72 (1.55-1.91) for those who were obese (BMI ≥30 kg/m^2^). The linear trend was significant for all results (p<0.001) (10). Another cohort study came to a similar conclusion that BMI was an independent risk factor for stroke in both women and men (28). However, some studies have reported no association between BMI and stroke incidence (14, 38). In addition, the results of our study with a multivariate-adjusted model using BMI classification as the independent variable showed an HR of 1.261 for normal-weight participants using underweight participants as a reference, but there was no statistical difference. This implies that underweight participants had no increased risk of stroke compared to normal-weight participants. However, overweight and obese participants had HRs of 1.434 and 1.546, respectively, which were statistically different. Similar results were obtained in the male and female populations. Results from a cohort study in Japan showed a significantly higher risk of stroke in men with a BMI ≤18.5 kg/m^2^ compared with normal-weight men (HR 2.11, 95% CI: 1.17-3.82) (17). Another study showed an increased risk of hemorrhagic stroke in women with low body weight (<20 kg/m^2^) compared with patients with a BMI of 20-22.4 kg/m^2^ (HR 1.90, 95% CI: 1.22-2.95) (23). However, other studies have obtained the opposite result, i.e., underweight participants had a lower risk of stroke than normal-weight participants (10, 28, 37, 39). Thus, although it is well established that being overweight or obese is considered a risk factor for stroke, the relationship between underweight and stroke remains controversial. Our findings showed that under-weight participants did not have a significantly increased risk of stroke compared to normal-weight participants, both in the overall population and in males or females. The possible reasons for these inconsistent findings are differences in the sex ratio, time of implementation, BMI range, and adjustment factors of these studies. Our study strengthened the argument that a high BMI positively associates with the risk of new-onset stroke in current literature. Our analysis also confirmed that being underweight does not reduce stroke risk in the Chinese middle-aged and elderly population. Previous studies have found that good stroke prevention management should include identifying and treating stroke risk factors and that good lifestyle management can reduce the burden of hospital care and reduce reliance on emergency services (40–43). This informs stroke prevention that weight loss through lifestyle management can reduce stroke risk but that too low a weight does not provide additional benefits. Second, compared with other studies, the independent variable in our study used both BMI as a categorical variable and a continuous variable of BMI to explore its relationship with stroke risk, which reduced the loss of information and quantified their relationship. In addition, we explored a possible non-linear relationship between BMI and stroke risk.

It is noteworthy that the results of the multivariate adjustment model after classifying BMI showed an overall increasing trend in HR from the normal weight group to the obese group, using the underweight participants as a reference, but the results were similar for the overweight and obese groups. That is, the trend of increasing HR values from the underweight group to the overweight group was evident and terminated in the range of overweight to obese. This suggests that there may be a non-linear relationship between BMI and the occurrence of stroke. Second, it is worth considering that conclusions based on linear regression analysis may be affected by the non-linear relationship, leading to differences in the fitted linear relationship. In other words, there may be a non-linear relationship between BMI and stroke incidence, which may also account for the discrepancy in the results of the studies mentioned above. In this study, we used Cox proportional hazards regression with cubic spline function and smoothed curve fitting to test our hypothesis. Ultimately, we observed a non-linear relationship between BMI and stroke risk, with an inflection point of 26.63 kg/m^2^ for BMI.

In addition, the non-linear connection may also be different for men and women owing to variations in body fat percentage and distribution (44). Therefore, we stratified participants by gender to further explore the association between BMI and stroke risk. We found a saturation effect curve between BMI and the risk of stroke in men, with an inflection point of 25.94 kg/m^2^ for BMI. In contrast, no non-linear relationship was found between BMI and stroke in female participants. This facilitates clinical consultation and provides a decision-making framework to optimize stroke prevention. When BMI is greater than 25.94 kg/m^2^ in men, weight loss alone does not substantially reduce stroke risk and may need to be combined with control of other risk factors such as hyperlipidemia, hypertension, diabetes, and smoking. In men, the risk of stroke may be significantly reduced by controlling BMI below 25.94 kg/m^2^.

It is yet unknown why there is a non-linear link between BMI and the risk of stroke. We found that individuals with BMI ≤ 26.63 kg/m^2^ had lower TC, FPG, LDL-c, TG, UA, and SBP than those with BMI>26.63 kg/m^2^. In addition, those with a BMI ≤26.63 kg/m^2^ had lower rates of diabetes and smoking (**Supplementary Table S2**). However, these indicators are closely related to the risk of stroke (45–49). Due to the existence of these risk factors, the effect of BMI on stroke is relatively weak when BMI is greater than 26.63kg/m^2^. On the contrary, in people with BMI less than 26.63kg/m^2^, these stroke risk factors are fewer and have a smaller impact on stroke, while the effect of BMI is relatively enhanced. This may be an important reason for the non-linear association. Furthermore, a non-linear relationship is one in which a change in one variable does not correspond to the same constant change in another variable. In other words, it may mean that the relationship between two variables is unpredictable. On the other hand, non-linear entities can be interrelated in a predictable but more complex way than linear entities. Given the complexity of the relationship between BMI and stroke, the non-linear relationship may also be closer to their true relationship. Furthermore, the distribution and proportions of body fat varied between men and women (44), which may account for the distinct associations between BMI and stroke risk shown in our research for men and women.

### Study strengths and limitations

We enumerated the following strengths of our study. First, the independent variables in our study employed both categorical and continuous BMI to evaluate its association with the risk of stroke, reducing information loss and quantifying their relationship. Second, Missing data were handled using multiple imputations. This approach can enhance statistical power and reduce potential bias brought on by the absence of covariate information. Third, our study is a significant improvement compared to previous studies regarding the nonlinearity addressing; for the first time, we found a non-linear association between BMI and stroke risk in men. In addition, we ran a battery of sensitivity analyses to ensure the stability of our finding (reanalyzing the non-linear association between BMI and stroke incidence after excluding participants with diabetes, hypertension, and CKD and calculating E-values to investigate the possibility of unmeasured confounding variables).

Potential limitations should be noted. First, the population included in this study was middle-aged and elderly Chinese; therefore, the generalizability of these results to younger populations and other ethnicities needs further validation. In the future, we will collaborate with researchers from outside of China to confirm the associations in populations with distinct genetic backgrounds. Second, BMI was only assessed once, at baseline, and not again thereafter. Their changes over time were disregarded; in addition, certain indicators associated with stroke were missing from the original data, including waist-to-hip ratio, medications, and genetic history of stroke. We can also consider structuring our studies or collaborating with other researchers to collect as many data points as possible, such as information on how BMI varies during participants’ follow-up. Third, stroke diagnosis relies on self-reported stroke history, and the subtypes and stages of stroke are unclear. In the future, we will need to combine medical history information and imaging findings to diagnose stroke. Fourth, as with all observational research, uncontrolled or unmeasured confounders may still exist even if known potential confounders are adjusted for. However, we estimated E-values to assess the possible influence of unmeasured confounders and discovered that they were unlikely to explain and influence our study’s results. Finally, this is an observational study that does not prove a causal relationship between BMI and stroke risk; rather, it only establishes an association between them.

## Conclusion

In the Chinese middle-aged and elderly population, BMI had a specific non-linear association and saturation effect with stroke risk in men but a linear association in women. In men, the inflection point for BMI was 25.94 kg/m^2,^ and there was a significant positive association between BMI and stroke risk when BMI was below the inflection point. When BMI was ≥ 25.94 kg/m^2^, their relationship was not statistically different; weight loss alone does not substantially reduce stroke risk and may need to be combined with control of other risk factors such as hyperlipidemia, hypertension, diabetes, and smoking. By controlling BMI below 25.94 kg/m^2^ in men, further BMI decline may significantly reduce stroke risk. In the future, ethnically diverse, larger multicenter prospective studies are needed to validate our findings.

## Data availability statement

The datasets presented in this study can be found in online repositories. The names of the repository/repositories and accession number(s) can be found below: Data are available from http://www.isss.pku.edu.cn/cfps/. Follow the prompts to register as a user and download the data once it has been reviewed and approved.

## Ethics statement

The Biomedical Ethics Review Committee of Peking University gave its approval to this study, which was carried out ethically and in compliance with the Helsinki Declaration. Additionally, written informed consent has been obtained from all participants (IRB00001052–11015). The patients/participants provided their written informed consent to participate in this study.

## Author contributions

GW, FL, CC, and YH conceived the research, drafted the manuscript, and performed the statistical analysis. YH and HH revised the manuscript and designed the study. All authors contributed to the article and approved the submitted version.
