# Supplementary material for: Non-linear dose-response relationship between body mass index and stroke risk in middle-aged and elderly Chinese men: a nationwide Longitudinal Cohort Study from CHARLS
Source: Front Endocrinol (Lausanne). 2023 Jul 5;14:1203896. doi: 10.3389/fendo.2023.1203896 (PMC10356587; doi:10.3389/fendo.2023.1203896)
Supplement: Supplementary file 1 [file Table_1.docx]

**Non-linear dose-response relationship between body mass index and stroke risk in middle-aged and elderly Chinese men: A nationwide Longitudinal Cohort Study from CHARLS**

**Running title: BMI and stroke**

**Gang Wei^1#^, Feng Lin^1#^, Changchun Cao^2#^, Haofei Hu^3*^, Yong Han^4*^**

^1^Department of Emergency, Hechi People's Hospital, Hechi 547000, Guangxi Zhuang Autonomous Region, China.

^2^Department of Rehabilitation, Shenzhen Dapeng New District Nan'ao People's Hospital, Shenzhen 518000, Guangdong Province, China.

^3^Department of Nephrology, Shenzhen Second People's Hospital, Shenzhen 518035, Guangdong Province, China.

^4^Department of emergency, Shenzhen Second People's Hospital, Shenzhen 518035, Guangdong Province, China

Gang Wei^1#^, Feng Lin^1#^, and Changchun Cao^2#^ have contributed equally to this work.

*Corresponding author

Haofei Hu

Department of Nephrology, Shenzhen Second People's Hospital

No.3002 Sungang Road, Futian District,

Shenzhen 518035,

Guangdong Province,

China

E-mail: haofeihu0319@126.com

*Corresponding author

Yong Han

Department of Emergency, Shenzhen Second People's Hospital

No.3002 Sungang Road, Futian District,

Shenzhen 518035,

Guangdong Province,

China

E-mail: hanyong511023@163.com

Table S1 Collinearity screening

|  | Step 1 | Step 2 |
| --- | --- | --- |
| BMI | 1.3 | 1.3 |
| Age | 1.2 | 1.2 |
| sex | 2.3 | 2.3 |
| WBC | 1.2 | 1.2 |
| PLT | 1.2 | 1.2 |
| BUN | 1.2 | 1.2 |
| FPG | 2 | 2 |
| Scr | 1.5 | 1.5 |
| TC | 14.7 | NA |
| TG | 8.1 | 1.6 |
| HDL-c | 3.4 | 1.4 |
| LDL-c | 12.4 | 1.2 |
| CrP | 1.1 | 1.1 |
| HBA1c | 1.9 | 1.9 |
| UA | 1.4 | 1.4 |
| HCT | 1.9 | 1.9 |
| HB | 1.9 | 1.9 |
| SBP | 1.3 | 1.3 |
| DBP | 1.2 | 1.2 |
| Hypertension | 1.3 | 1.3 |
| Diabete | 1.2 | 1.2 |
| Malignant tumors | 1 | 1 |
| CLD | 1 | 1 |
| CHD | 1.1 | 1.1 |
| CKD | 1 | 1 |
| Mental disease | 1 | 1 |
| Smoking status | 1.8 | 1.8 |
| Drinking status | 1.1 | 1.1 |

Variables excluded from collinearity screening: TC

SD,standard deviation ; n, number (%);

BMI, body mass index; PLT, platelet; TG, triglyceride; HGB, hemoglobin concentration; TC, total cholesterol; HCT, hematocrit; LDL-c, low-density lipoproteins cholesterol; Scr, serum creatinine; HDL-c, high-density lipoprotein cholesterol; CKD, Chronic kidney diseases;BUN, blood urea nitrogen; ALT, alanine aminotransferase; AST, aspartate aminotransferase; CLD, Chronic Lung Diseases; CHD, coronary heart disease.

**Table S2 The Baseline Characteristics of participants on both sides of the inflection point**

| BMI qroups | <26.63 | >=26.63 | P-value |
| --- | --- | --- | --- |
| N | 9341 | 2820 |  |
| Age(years, mean ± SD) | 59.45 ± 9.82 | 56.82 ± 8.80 | <0.001 |
| PLT (10^9/L, mean ± SD) | 210.38 ± 72.87 | 216.60 ± 72.22 | <0.001 |
| BUN (mmol/L, mean ± SD) | 15.81 ± 4.63 | 15.37 ± 4.41 | <0.001 |
| FPG (mg/L, mean ± SD) | 108.02 ± 35.46 | 116.42 ± 40.49 | <0.001 |
| Scr(mg/dL, mean ± SD) | 0.78 ± 0.24 | 0.77 ± 0.21 | 0.004 |
| TG (mg/dL, median, quartile) | 191.59 ± 36.17 | 199.75 ± 40.25 | <0.001 |
| TG (mg/dL, median, quartile) | 122.87 ± 95.82 | 169.31 ± 141.79 | <0.001 |
| HDL-c(mg/dL, mean ± SD) | 53.04 ± 15.27 | 45.44 ± 13.19 | <0.001 |
| LDL-c(mg/dL, mean ± SD) | 114.70 ± 33.89 | 119.82 ± 37.40 | <0.001 |
| CRP (mg/L, median, quartile) | 2.60 ± 7.59 | 2.74 ± 6.32 | 0.375 |
| HBA1C (%,mean ± SD) | 5.21 ± 0.76 | 5.41 ± 0.89 | <0.001 |
| UA (mg/dL, mean ± SD) | 4.39 ± 1.23 | 4.57 ± 1.25 | <0.001 |
| HGB(g/L, mean ± SD) | 14.23 ± 2.22 | 14.51 ± 2.24 | <0.001 |
| SBP (mmHg, mean ± SD) | 129.13 ± 28.23 | 137.45 ± 33.82 | <0.001 |
| DBP (mmHg, mean ± SD) | 73.75 ± 13.20 | 79.29 ± 14.35 | <0.001 |
| Diabetes | 363 (3.89%) | 288 (10.21%) | <0.001 |
| Malignant tumors(n.%) | 79 (0.85%) | 46 (1.63%) |  |
| CLD (n, %) | 994 (10.64%) | 247 (8.76%) | 0.004 |
| CHD (n, %) | 897 (9.60%) | 443 (15.71%) |  |
| CKD (n, %) | 593 (6.35%) | 170 (6.03%) | 0.539 |
| Mental disease (n, %) | 108 (1.16%) | 30 (1.06%) | 0.685 |
| Smoking status (n, %) |  |  | <0.001 |
| Never | 5435 (58.18%) | 2007 (71.17%) |  |
| Ever | 760 (8.14%) | 262 (9.29%) |  |
| Current | 3146 (33.68%) | 551 (19.54%) |  |
| Drinking status |  |  | <0.001 |
| Never | 1295 (13.86%) | 349 (12.38%) |  |
| Ever | 5582 (59.76%) | 1920 (68.09%) |  |
| Current | 2464 (26.38%) | 551 (19.54%) |  |

SD,standard deviation ; n, number (%);

BMI, body mass index; PLT, platelet; TG, triglyceride; HGB, hemoglobin concentration; TC, total cholesterol; HCT, hematocrit; LDL-c, low-density lipoproteins cholesterol; Scr, serum creatinine; HDL-c, high-density lipoprotein cholesterol; CKD, Chronic kidney diseases;BUN, blood urea nitrogen; ALT, alanine aminotransferase; AST, aspartate aminotransferase; CLD, Chronic Lung Diseases; CHD, coronary heart disease.

**Table S3 Relationship between BMI and the risk of stroke analyzed by two-piecewise linear regression model in different age subgroups.**

| Age groups | Age<60 | 60 to <70 | ≥70 | Total |
| --- | --- | --- | --- | --- |
| Fitting model by standard linear regression | 1.028 (1.006, 1.051) 0.012 | 1.033 (1.006, 1.059) 0.0142 | 1.006 (0.972, 1.041) 0.738 | 1.026 (1.011, 1.041) <0.001 |
| Fitting model by two-piecewise linear regression | |  |  |  |
| Inflection points of BMI (kg/m^2^) | 26.94 | 29.79 | 17.62 | 26.625 |
| ≤ Inflection point | 1.065 (1.027, 1.104) <0.001 | 1.057 (1.023, 1.092) <0.001 | 0.643 (0.449, 0.920) 0.016 | 1.045 (1.020, 1.070) <0.001 |
| > Inflection point | 0.982 (0.937, 1.029) 0.450 | 0.906 (0.793, 1.035) 0.147 | 1.020 (0.985, 1.056) 0.274 | 0.999 (0.968, 1.031) 0.947 |
| P for log-likelihood ratio test | 0.016 | 0.018 | 0.025 | 0.047 |

Note: In all age subgroups, we adjusted age, sex, CRP, HGB, LDL-c, HDL-c, TG, FPG, HCT, HBA1c, hypertension, diabetes, SBP, CKD, CHD, mental disease, CLD, drinking status, smoking status.
